# A nationwide cross-sectional survey of student experiential practice at community pharmacies in South Korea

**DOI:** 10.1186/s12909-019-1879-1

**Published:** 2019-12-02

**Authors:** Yejee Kim, Kyeong Hye Jeong, EunYoung Kim

**Affiliations:** 1 Clinical Data Analysis, Evidence Based Clinical Research Lab., Department of Health Science and Clinical Pharmacy, College of Pharmacy, Chung-Ang University, 84 Heukseok-Ro, Dangjak-gu, Seoul, 06974 Republic of Korea; 2 College of Pharmacy, Chung-Ang University, Seoul, 06974 Republic of Korea; 3 Division of Licensing of Medicines and Regulatory Science, The Graduate School Pharmaceutical Management, Chung-Ang University, 84 Heukseok-Ro, Dangjak-gu, Seoul, 06974 Republic of Korea

**Keywords:** Community pharmacy experiential practice (CPEP), Students, CAPE, CPEPM, Survey

## Abstract

**Background:**

In South Korea, community pharmacy experiential practice (CPEP) is very important because most pharmacists (71.8%) work in community pharmacies, which also employ the majority of students after graduation. The present study investigated student responses to the current CPEP status, suggestions for improvement, and advancement in their competency after practice based on evaluation of Community Pharmacy Experiential Practice Model (CPEPM) outcomes.

**Methods:**

A nationwide cross-sectional, self-administered online survey was conducted in 2017 for the sixth-year pharmacy students who completed CPEP, using 50 item questionnaire. The answers were evaluated using the 4-point Likert scale, used a scoring system from 1 (strongly disagree) to 4 (strongly agree). Responses of 1 and 2 were considered negative, and 3 and 4 were considered positive. To identify factors affecting CPEPM outcomes, multivariate linear regression analysis was performed.

**Results:**

Initially, 1138 students participated in the survey. Of these, responses from 492 students were excluded due to missing data and eventually, data from 646 students were included in the analysis. In total, 95% of students responded in the affirmative that practical training influenced their future career decision. In addition, 78.5% of students were satisfied with the training. Further, they responded that their ability improved based on CPEPM outcomes. The most positive capability change was in the subdomain “personal and professional development”, followed by “inter professional collaboration”.

**Conclusions:**

Students responded with the higher rating for satisfaction factor, who regarded CPEP as helpful in future career decision making, and those who wanted to practice elective Advanced Pharmacy Practice Education at a community pharmacy had a positive CPEPM outcome, while age was found to be a negative factor in terms of the regression analysis. These are valuable findings as they represent the current student perception of CPEP nationwide. They provide a basis to improve the quality of CPEP-based education not only in Korea, but in other countries as well.

## Background

Pharmacy education in Korea has drastically changed since 2009; the initial four-year program has changed to a six (two + four)-year program and the number of pharmacy schools have increased from 20 to 35. These changes were made for academic improvement in the pharmacy sector [1]. The biggest change in the (two + four) year program is that students have to complete 1 year of experiential practice as part of the core curriculum.

During the training, students have to complete 200 h of core Advanced Pharmacy Practice Education (APPE). In addition, students can elect to participate in 600 h of elective APPE at community pharmacies. The core APPE classes are scheduled depending on the convenience of the school, and most schools hold them in the first semester of the sixth year [2]. Approximately 1600 students participate in community pharmacy experiential practice (CPEP) each year [3].

In Korea, there are 68,616 pharmacists and half of them are active [4]. There are 21,737 pharmacies in Korea per the Health Insurance Review & Assessment Service (HIRA) data in 2017 [5]. Among the active pharmacists, 71.8% work in community pharmacies; a desired workplace for the majority of students after graduation [6]. Community pharmacies are thus, one of the most important practical sites for pharmacy students’ education.

Evaluation of students via the most current practical experience is an important indicator of the level and quality of education and is the most useful basis for evaluating curriculum [7]. Although there have been several studies on practical education of community pharmacy students in Korea, these have been in the initial stage of practical training, limited to specific geographical areas, or have used small sample sizes; thus, they have not reflected contemporary opinions of students across the country [7–10]. A recent study investigated the responses of pharmacy students over 3 years regarding the current status of experiential education [7]. This study was not representative of the country as it was performed in only one city. It is therefore essential to investigate nationwide student opinion regarding CPEP.

Further, in terms of outcome evaluation, assessment of student responses after practice is also essential to evaluate the level of practice quality [11]. There are several studies regarding outcome evaluation following CPEP in other countries, but no such domestic study has been reported [12–14].

The purpose of the present study was to assess student responses to the current status of CPEP, suggestions for improvement, and evaluate advancement in their competency following practice based on community pharmacy experiential practice model (CPEPM) outcomes.

## Methods

The study consisted of a nationwide cross-sectional, self-administered, online survey conducted in 2017. The study population was the sixth year pharmacy students who had completed core APPE at community pharmacies in South Korea in 2017; the number of students enrolled that year was 1600 [3].

The survey was conducted for 4 months beginning from June, when most schools completed the core APPE. The survey instrument is attached as Additional file 1: Appendix 1. A KPA representative called the student president in each school nationwide. The representative explained the background of the study to them, and with the student president’s agreement, an e-mail containing a link to the online survey (Now & Survey, Co in Seoul, South Korea) was sent to each of them. Each school’s president then distributed the survey link via the popular private social media network service “Kakao talk” [14]. Follow-up notifications were sent twice every other week to improve the response rate per the modified Dillman Method [15]. To protect the respondents’ privacy, no identifiable information was included in the completed questionnaires, and thus the survey was kept anonymous.

The initial survey instrument was developed based on a previous study, the “Essential Practice White Paper”, and the “Community Pharmacy Essential Practice Manual” published by the Korean Association of Pharmacy Education (KAPE) and the Center for the Advancement of Pharmacy Education (CAPE) outcomes [2, 16, 17].

After the initial draft of the survey was prepared, face validation was done with a small group of preceptors and students. Then, two clinical pharmacy professors who had experience as experiential education coordinators modified the questionnaire. The revised questionnaire was pilot-tested by three students, and the final questions were confirmed. In particular, the survey items measuring changes in a student’s competency after CPEP (CPEPM outcome) were selected from the “Community Pharmacy Essential Practice Manual”, which were comparable to those in CAPE outcomes” [16–18].

The survey addressed demographics (5 questions), general CPEP status and evaluation (23 questions), suggestions for improvement (5 questions), and outcomes in terms of competency changes after CPEP (17 questions).

The answers were evaluated using the 4-point Likert scale, scoring from 1 (strongly disagree) to 4 (strongly agree). Responses of 1 and 2 were considered negative, while 3 and 4 were considered positive [19]. The CPEPM outcome was measured based on 17 sub-items questions regarding the student’s competency changes after CPEP. The CPEPM outcome was calculated as the mean sum of responses to those questions.

Reliability analysis was conducted on each survey item. Internal consistency reliabilities measured with Cronbach’s alpha values of satisfaction (0.871), stress (0.816), changes in competency (0.926), and evaluation (0.774) were acceptable. The items were randomized and rotated in each analysis to reduce response bias. Although all sixth-year pharmacy students who had completed core APPE at community pharmacies in 2017 were targeted, this survey was voluntary and self-administered. Thus, information regarding the basic demographic variables of all students enrolled in 2017 were collected to show that the sample was approximately similar to the population of students [20, 21].

During the statistical analysis, a descriptive analysis was performed to summarize the item responses. Chi-square and t-tests were used to identify any differences between the student responses regarding demographics, perceptions (positive, negative), and differences in the CPEPM outcome.

To identify factors affecting CPEPM outcome in students after practice, univariate analysis was performed, followed by multivariate linear regression analysis using statistically significant variables, in addition to the data regarding the gender and practice sites [22, 23]. CPEPM outcome was taken as a dependent variable. The independent variables were demographics (age, gender, practice sites) and perception (CPEP was performed systematically; CPEP was helpful in future career decision; stress increased during CPEP; stress factor; satisfaction; satisfaction factor; plan to do elective APPE at community pharmacy). Statistical analysis was done using SPSS Version 23 (SPSS, Inc., Chicago, IL). Statistical significance was set at *P* < 0.05 in two-tailed tests.

## Results

Initially, 1138 students out of a total 1600 students enrolled in 2017 in the sixth-year (response rate (RR): 71.1%) participated in the survey [3]. However, data from 492 students were excluded due to incomplete surveys with missing information. As a result, the data from a total of 646 students (RR: 40.4%, 646/1600) were analyzed. The demographics are listed in Table 1. Students participated nationwide and were evenly distributed between metropolitan and provincial areas. Although we attempted to collect information regarding the basic demographics of all students enrolled in 2017, in order to demonstrate the similarity between the sample and the general student population, none of these variables were available, except for gender [20, 21]. In this study, 61.0% of participants were female, which is not statistically different from gender distribution of all school of pharmacy sixth-year students (58.3%) in 2017 (*p* = 0.256).

**Table 1:** Demographics of Students (n = 646)

Variables	Students (n = 646)
	Number (%)
Age	
< 25	128 (19.8)
25~29	423 (65.5)
30~34	81 (12.5)
≥ 35	14 (2.2)
Gender	
Female	394 (61.0)
Male	252 (39.0)
Practice site	
Seoul and Metropolitan	341 (52.8)
Gyeonggi-do	87 (13.5)
Gangwon-do	7 (1.1)
Chungcheong-do	60 (9.3)
Jeolla-do and Jeju-do	80 (12.4)
Gyungsang-do	71 (11.0)
Factors affecting practice site selection	
Transportation time (distance to home)	272 (42.1)
College policy (random assignment)	266 (41.2)
Awareness of pharmacy	87 (13.5)
Other	21 (3.3)
Items sold by the pharmacy	
Functional health food	629 (97.4)
Medical devices	301 (46.6)
Animal medicine	297 (46.0)
Cosmetics	268 (41.5)
Oriental medicine	149 (23.1)

Most students in the survey (85.3%) were less than 30 years old.

**Fig. 1:**
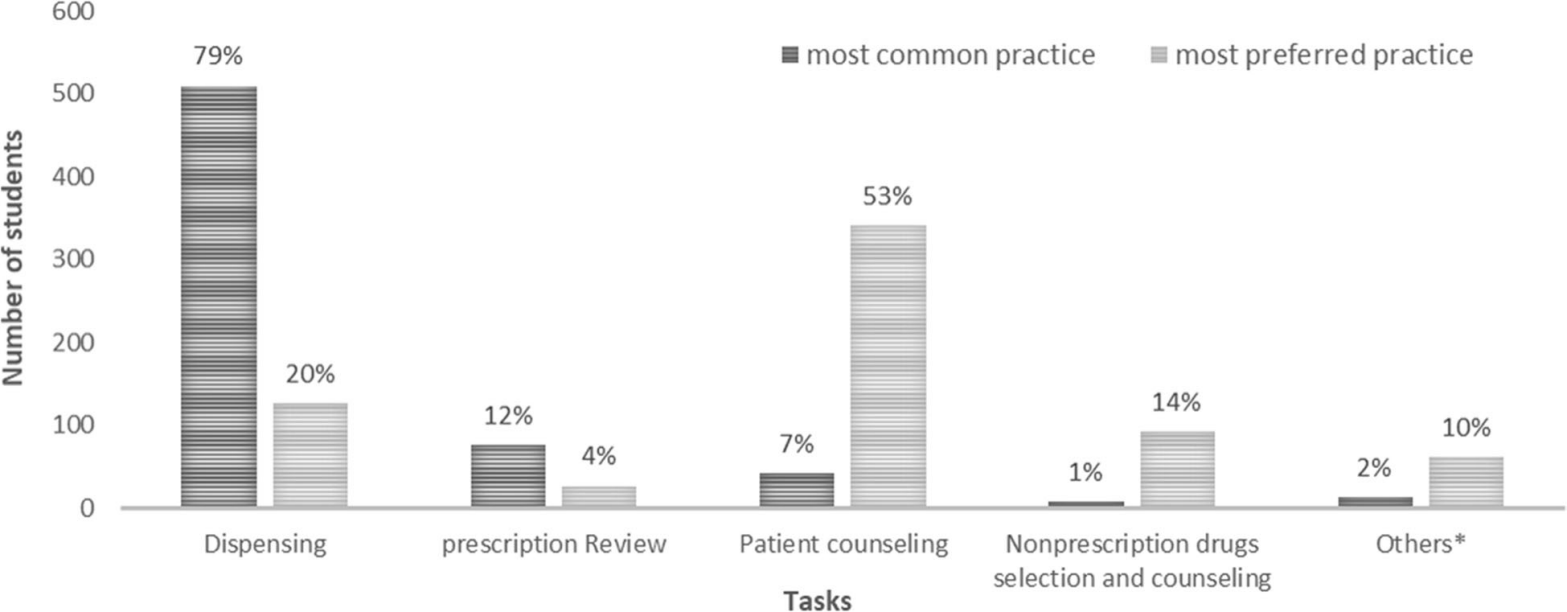
Most common and most preferred student practice tasks during CPEP (*n* = 646). *Others: oriental medicine, functional health food, medical devices and quasi drugs [24], and pharmacy management and insurance claim

The tasks performed by the students and the most preferred ones are shown in Fig. 1. The most commonly performed task was dispensing, whereas the most preferred task was patient counseling.

Student responses regarding the general status and evaluation of CPEP are shown in Table 2.

**Table 2:** Status and Evaluation of Community Pharmacy Experiential Practice (n = 646)

Survey Questions	Number (%)
Most positive aspect of experiential education	
Help in navigating future career	209 (32.4)
Help in improving professionalism as an entry-level pharmacist	157 (24.3)
Opportunities to learn various areas not taught in school	149 (23.1)
Knowledge gained in school can be linked to real life	75 (11.6)
Opportunity to understand the role of the community pharmacist	56 (8.7)
The site of practice pharmacy was well-organized and systematically performed experiential practice.	
Yes (positive)	481 (74.5)
No (negative)	165 (25.6)
Community pharmacy training was helpful in career decision-making	
Yes (positive)	613 (94.9)
No (negative)	33 (5.1)
Criteria for evaluation of the practical training was clearly presented	
Yes (strongly agree, agree)	336 (52.0)
No (strongly disagree, disagree)	310 (48.0)
The preceptor clearly understood the assessment methods and standards	
Yes (strongly agree, agree)	416 (64.4)
No (strongly disagree, disagree)	230 (35.6)
The preceptor’s evaluation was fair	
Yes (strongly agree, agree)	487 (75.4)
No (strongly disagree, disagree)	159 (24.6)
Plans to undergo elective APPE in a community pharmacy	
Yes (strongly agree, agree)	209 (32.4)
No (strongly disagree, disagree)	437 (67.6)

In general, students positively evaluated community pharmacy practical training. About 95% of the students responded affirmatively that CPEP had helped them make future career decisions. Almost 75% of the students answered that the practical training was well organized and conducted systematically. Over 75% of students responded that they believed their preceptor evaluated them fairly, though only 50% of the students agreed that the evaluation criteria were clearly presented.

**Fig. 2:**
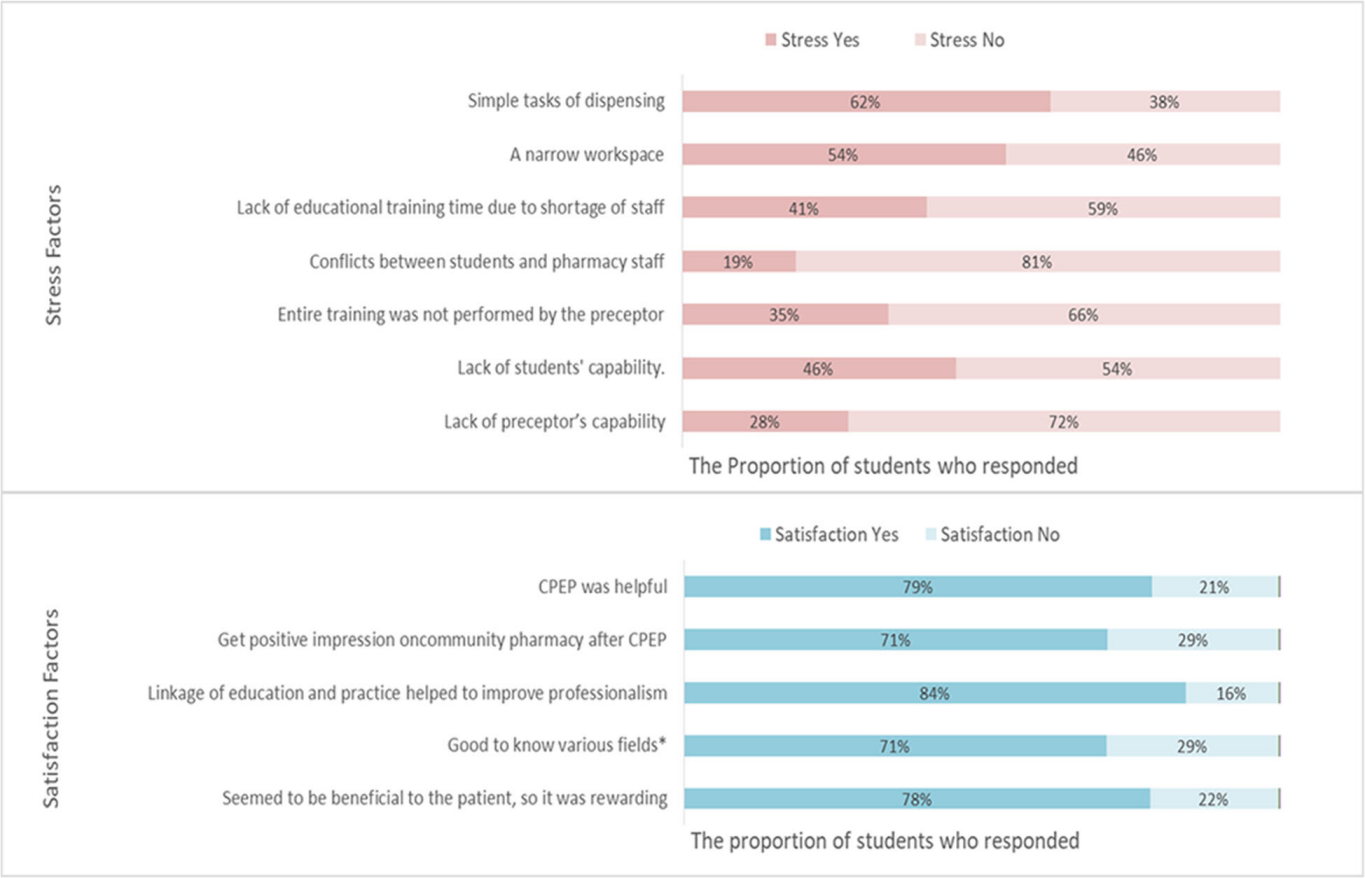
Factors related to Satisfaction and Stress during Community Pharmacy Experiential Practice (n = 646). * OTC, Oriental medicine, medical device, cosmetics

Student responses regarding stress and satisfaction during CPEP are shown in Fig. 2.

The majority of students (57%) stated that the training did not increase stress. The most common stress-inducing factors were the simple tasks of dispensing, followed by the use of a narrow workspace.

Students were asked if they were satisfied with CPEP, and 78.5% of them responded positively. The top-ranked item associated with satisfaction was “linkage of education and practice helped in improving professionalism”, followed by “CPEP was helpful” and “a positive impression regarding community pharmacy after CPEP”.

**Fig. 3:**
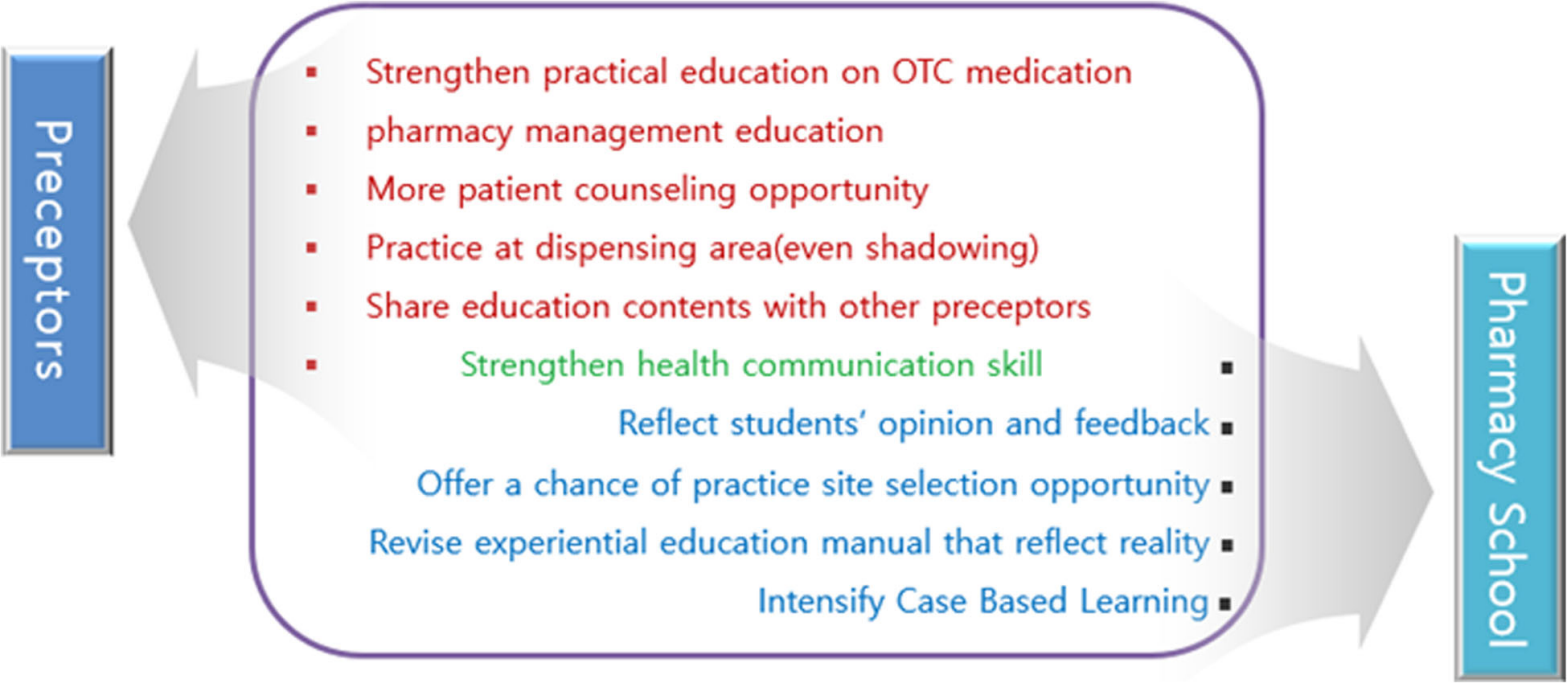
Suggestions for improvement of students regarding the Community Pharmacy. Experiential Practice to the preceptors and school of pharmacy (n = 646)

Students responded to five multiple choice questions about their opinion on how to improve CPEP (Additional file 2: Appendix 2). Among the opinions supported by more than 20% of the answers for each question, the proposal for the school and the proposal for the preceptor group are separately shown in Fig. 3.

### Community pharmacy experiential practice model (CPEPM) outcomes

CPEPM outcomes represent whether the capability of students changed after experiential education in a community pharmacy, and the responses are summarized in Table 3.

**Table 3:** Capability improvement after community pharmacy practical training evaluated via CPEPM outcomes (n = 646)

Items in CPEPM	CAPEM related CAP E[18] Domain^a^	positive % (N)	Negative % (N)	Mean^b^ ± SD
Identify problems related to the patient’s medications and suggest solutions	2.1, 3.1	65.3 (422)	34.7 (224)	2.70 ± 0.71
Monitor the patients’ medication effects, compliance and adverse effects, adjust care plan as needed.	2.1, 3.1	61.3 (396)	38.7 (250)	2.65 ± 0.77
Collect subjective and objective evidence related to the patient, by performing patient assessment from charts, pharmacist records, and patient/family interviews, then recommend optimal pharmacotherapy.	2.1, 3.1	58.6 (379)	41.3 (267)	2.60 ± 0.77
Optimizing the medication use system (i.e., Create, manage, and properly dispose of pharmacy documents)	2.1	48.6 (314)	51.4 (332)	2.44 ± 0.80
Effectively manage procurement, sale, storage, and inventory control of medication and pharmacy items	2.2	58.5 (378)	41.5 (268)	2.60 ± 0.83
Understand work priorities, and carry out tasks systematically	2.2	79.2 (512)	20.7 (134)	2.91 ± 0.67
Provide adequate counseling and education on pharmacotherapy, non-pharmacotherapy, and preventive therapy	2.3	64.4 (416)	35.6 (230)	2.70 ± 0.73
Provide health education to community residents about issues including health promotion, disease prevention, drug abuse prevention.	2.3	55.8 (360)	44.2 (286)	2.56 ± 0.77
Identify and solve problems arising during training	3.1	73.8 (477)	26.2 (169)	2.79 ± 0.65
Create appropriate presentation materials and deliver it properly to the intended audience	3.2	78.8 (509)	21.2 (137)	2.91 ± 0.67
Empower patients to take responsibility for, and control of, their health.	3.3	74.5 (481)	25.5 (165)	2.83 ± 0.66
Advise patients to obtain the resources and care required in an efficient and cost-effective manner	3.3	66.3 (428)	33.8 (218)	2.70 ± 0.73
Demonstrate mutual respect (preceptor, colleague) and values of co-operation to meet patient care needs	3.4	93.5 (604)	6.5 (42)	3.25 ± 0.64
Effectively communicate with physicians and resolve medication-related problems	3.6	69.0 (446)	31.0 (200)	2.77 ± 0.74
Practice communication skills through effective verbal communication	3.6	83.7 (541)	16.2 (105)	3.01 ± 0.67
-Experience of and understanding of the roles and responsibilities of pharmacists	4.4	93.9 (607)	6.1 (39)	3.27 ± 0.63
-Realization of the importance of improving competency as a professional through practical training	4.4	94.7 (612)	5.2 (34)	3.42 ± 0.64

Score: 1 (Strongly disagree), 2 (Disagree), 3 (Agree), 4 (Strongly agree), Positive (Agree, Strongly agree), Negative (Strongly disagree, Disagree)

*SD* standard deviation, *CPEPM* Community Pharmacy Experiential Education Manual, *CAPE* Center for the Advancement of Pharmacy Educatio

^a^ Domain 2 (Essentials for Practice and Care): 2.1 (Patient-centered care), 2.2 (Medication use system management), 2.3 (Health and wellness promoter)

Domain 3 (Approach to Practice and Care): 3.1 (Problem solving), 3.2 (educator), 3.3 (Patient advocacy), 3.4 (Inter professional collaboration), 3.6 (Communication),

Domain 4 (Personal and Professional Development): 4.4 (Professionalism)

^b^ Mean Score

Responding to 17 questions, the majority of students stated that their self-perceptions about capability changes improved after the experiential practice in all aspects, except in optimizing the medication use system, which only 48.6% responders found improved. Positive responses included “agree” or “strongly agree,” while negative responses included “strongly disagree” or “disagree”. The item with the most positive responses was “realization of the importance of improving competency as a professional (94.7%)”, followed by “experience and understanding of the role and responsibility of a pharmacist (93. 9%)”, and finally “values of mutual respect and co-operation to meet patient care needs (93.5%)”. In general, the students responded with high scores in conceptual areas such as understanding and realization, and with lower scores in areas needing direct action such as problem identification, consulting, and monitoring.

Multivariate regression analysis of changes in student competency after practical training based on CPEPM outcomes is summarized in Table 4. The model did not violate the assumptions of multivariate regression (e.g. normality, linearity, homoscedasticity) and multicollinearity do not exist. Factors affecting CPEPM outcomes positively were “satisfied with CPEP”, “satisfaction factors”, “the experiential education was systematic”, “CPEP was helpful in future career decisions”, and “plans for elective APPE in a community pharmacy”. The “age” was a negative variable for the outcome. All variables were statistically significant (*P* < 0.05).

**Table 4:** Factors affecting CPEPM outcome^a^ changes (multivariate linear regression)

Model	Unstandardized coefficient	Standardized coefficient	*P*-value
Independent variable	B (95% CI)	Beta	
(Constant)	1.359 (0.967 to 1.751)		0.000
Age^*^	−0.013 (−0.022 to −0.003)	−0.079	0.009
Gender	−0.043(−0.099 to 0.013)	− 0.045	0.136
Practice site	−0.003(− 0.016 to 0.009)	−0.015	0.614
CPEP was performed systematically^*^	0.100 (0.051 to 0.150)	0.179	0.000
CPEP was helpful in future career decision^*^	0.110 (0.059 to 0.162)	0.149	0.000
Stress increased during CPEP	−0.037(−0.074 to 0.001)	−0.073	0.058
Stress factor^b^	−0.015(− 0.070 to 0.041)	−0.021	0.604
Satisfaction^*^	0.127 (0.075 to 0.179)	0.235	0.000
Satisfaction factor^c*^	0.332 (0.256 to 0.407)	0.264	0.000
Plan to do elective APPE at community pharmacy^*^	0.076 (0.017 to 0.134)	0.076	0.012

^a^ CPEPM outcome is the sum of the 17 survey questions, measured using a 4-point Likert scale and divided by 17

^b^ Stress factors is the sum of 7 survey questions, measured using a 4-point Likert scale and divided by 7

^c^ Satisfaction factors is sum of 5 questions measured using a 4-point Likert scale and divided by 5

^*^ Statistically significant independent variable (*P* < 0.05)

## Discussion

The present study represents the results of the first nationwide survey in Korea to analyze the current status and outcomes of CPEP since the implementation of the (two + four)-year program in pharmacy schools. A nationwide study on hospital pharmacy preceptors has been conducted in 2018 in Korea; however, no nationwide studies focused on students have been reported [25].

### Status of community pharmacy experiential practice (CPEP)

Four years have passed since the implementation of CPEP, and the present study results showed nearly all students (95%) responding that the CPEP had an impact on their career decisions. Three out of four students in the study responded positively to the above aspects of practical training. These results indicate that CPEP has been well-implemented, compared to the results from previous studies [8, 9].

The students responded that the most frequently completed task during CPEP was dispensing, even though the most preferred task was patient counseling (which has also been reported in other studies) [17, 26]. However, most preceptors tend to hesitate when providing patient counseling opportunities to students because some patients dislike the idea of receiving counseling from pharmacy school student. After obtaining the patient’s consent, the preceptor should allow students to counsel a patient to improve their abilities. In addition, a system and guidelines should be established when outlining the responsibilities of students during counseling. This will be consistent with the purpose of practical training and will further improve student satisfaction. Even though most of the students were satisfied with CPEP, and despite their perception that their capability had improved post-CPEP, most of the students (67.6%) stated that they did not plan to do an elective APPE at a community pharmacy.

Although there are several reasons for this, three mains reasons were discussed in the small group face validation. First, the lack of an elective APPE at a community pharmacy site: experiential practice was implemented recently in South Korea, and there are not enough preceptors at a community pharmacy who could supervise 15 weeks of elective APPE. Second, the policy of the pharmacy school: several pharmacy schools do not allow for elective APPE at a community pharmacy. Finally, the students’ preference of experience: since most of the students will work in pharmacies after graduation, they tend to pursue elective APPE in areas other than pharmacies that they would otherwise not experience.

Most student responses to stressors during CPEP outlined that the biggest cause of stress was “simple tasks of dispensing”, followed by a “pharmacy environment with a narrow space”. These problems have also been reported in previous studies [8, 9] This suggests a need for close cooperation between schools and preceptors to conduct CPEP in a balanced manner, to impart practice in other areas in addition to dispensing, as suggested by the Community Pharmacy Essential Practice Manual shown in Fig. 1.

To minimize the problem of narrow spaces, the schools should visit community pharmacy practice sites prior to training. Only pharmacies of the appropriate size should be used, and the number of students practicing in a single site should be limited. As the number of pharmacies participating in this training increases, it is necessary to further develop and improve new training sites that are appropriate for student practice.

Students have emphasized the need to enhance their health communication skills. As an integral part of health care, health communication is important in dealing with patients, and with other health care providers to provide safe, good quality, patient-centered care. However, most pharmacy colleges in Korea do not include health communication skills in their curriculum. Pharmacy faculty members and KAPE should consider integrating healthcare communication into the regular curriculums. Recently, the Korea Communication Association was launched, which encourages the use of published textbooks on healthcare communication [27].

Students have suggested the need for more case-based learning (CBL). The Korean schools of pharmacy education taught about the standardized treatments used for different diseases taught about the standardized treatments used for different disease; however, the (two + four)-year programs have gradually adopted CBL and problem-based learning (PBL) from the medical school education programs [28–31]. However, due to the lack of professors and financial support, CBL and PBL are not as active in pharmacy schools as they are in schools of medicine, and remain in early stages of deployment. Community pharmacies are good sites to practice CBL and PBL, allowing these education styles to be incorporated during the longer CPEP duration.

### CPEPM (community pharmacy experiential practice model) outcomes

The present study also highlights CPEPM outcomes, which are likely to provide CPEP educators with the ability to evaluate, reform, or implement the CPEP program [32, 33]. Experiential education in Korea encompasses one-fourth of the pharmacy curriculum. Experiential practice outcome assessment is an essential part of measuring the attitudes, skills, and knowledge of students in terms of the requirements of a qualified entry-level pharmacist [34]. Therefore, it will be useful to measure their capability in becoming a pharmacist [34–36]. The majority of students agreed that their capability improved through CPEP; in particular, their professionalism and “inter-professional collaboration” saw the best improvements. The positive responses to increase in professionalism were found to be higher in the present study compared to a previous US study [37]. The reason for this may be that in the US, pharmacy students are used to pharmacy practice beginning from the first year of study as a clerk or an intern pharmacist. However, most Korean students only have the first chance to work in a pharmacy during CPEP; thus, they have lesser exposure to the workings of a pharmacy. During training, interactions with their preceptors, dealing with the clinicians, and taking care of patients is likely to provide them with strong impressions about professionalism.

Student responses to identifying and assessing patient problems, monitoring, and providing adequate patient counseling and education were less positive than other responses in terms of the outcomes in the present study. These skills take time to learn and perfect, and thus need more PBL and CBL [38, 39]. Therefore, it is necessary to strengthen these exercises in school, in addition to the use of CPEP. A five-week practical duration may be insufficient to acquire the skills needed to solve the various health-related problems of patients in real life; the duration of the Korean core APPE is in fact shorter than those in other countries [11, 40, 41].

The least positive outcome in competency improvement was in optimizing the medication use system such as pharmacy document management (48%). Pharmacists usually complete this in the evening or on Saturday afternoon when they encounter fewer customers, however this is also post a student’s training time. Moreover, non-systematic implementation of Medicine Use Review (MUR), and Medication Therapy Management (MTM) in Korea has provided students with fewer opportunities to learn how to complete these tasks [42, 43].

Overall, the CPEPM outcome results show that the students had positive feelings about the improvement in their capabilities despite a short training period.

### Limitations and strengths

A limitation of the present study is that the survey may have had a selection bias because the respondents participated voluntarily. Therefore, it is possible that more students with stronger opinions participated.

Another major limitation is that there were a lot of missing data. This may have been due to two reasons. First, several domains were composed of multiple items (3~17 questions), and many students may have skipped some questions. Second, most of the survey period was during the summer break. To generalize this study to the entire Korean student population, the basic distribution of characteristics, including age, gender, and practice sites, among others should have been compared between this sample and the general student population. However, these data were not available, except for data regarding the gender distribution [20, 21]. Although, the number of respondents was much more than those in the nationwide preceptor survey of the United States or in the Korean KAPE survey, it is hard to eliminate the selection bias in this study, or to insist that these findings are likely to be similar across all students [2, 22]. Despite these limitations, this is the first study reporting a nationwide pharmacy student survey in Korea, and involved the largest number of students participating voluntarily from both metropolitan and provincial areas. Thus, it represents nationwide student opinion about community pharmacy practical training and improvements in their competency following the training.

## Conclusions

The majority of the students stated that their ability improved after experiential practice. Students responded with the higher rating for satisfaction factor, who regarded CPEP as helpful in future career decision making, and those who wanted to practice elective APPE at a community pharmacy had a positive CPEPM outcome, while age was found to be a negative factor in terms of the regression analysis. The students reported the least improvement in competency in the pharmacy document management area and expressed the need for more counseling opportunities and to strengthen their communication skills. More effort is needed to improve experiential pharmacy practice, especially considering these less successful CPEPM outcomes.

The information obtained from the present study provides a basis for improvement in pharmacy education in Korea, and also serves as a helpful reference for other countries when developing or planning new experiential education programs in community pharmacies.

## Supplementary information

**Additional file 1: Appendix 1.** Survey of a nationwide cross-sectional survey of student experiential practice at community pharmacies in South Korea

**Additional file 2: Appendix 2.** Suggestion for improvements in community pharmacy experiential practice

## Data Availability

The datasets used and/or analyzed to support the findings of the current study are available from the nationwide students’ survey of community pharmacy experiential practice conducted in 2017. The datasets analyzed during the current study are not publicly available. However, a copy of the survey was shown as a supplementary material file.

## Abbreviations

APPE: Advanced pharmacy practice education
CAPE: Center for the advancement of pharmacy education
CBL: Case based learning
CPEP: Community pharmacy educational practice
CPEPM: Community pharmacy educational practice model
HIRA: Health Insurance Review & Assessment Service
KAPE: Korean Association of Pharmacy Education
KPA: Korea Pharmaceutical Association
MTM: Medication therapy management
MUR: Medicines use review
PBL: Practice based learning
RR: Response rate
SCPEP: Standard of Community Pharmacy Experiential Practice
