# Supplementary material for: A nationwide cross-sectional survey of student experiential practice at community pharmacies in South Korea
Source: BMC Med Educ. 2019 Dec 2;19:445. doi: 10.1186/s12909-019-1879-1 (PMC6888916; doi:10.1186/s12909-019-1879-1)
Supplement: Supplementary file 1 — Additional file 1: Appendix 1. Survey of a nationwide cross-sectional survey of student experiential practice at community pharmacies in South Korea [file 12909_2019_1879_MOESM1_ESM.docx]

**Additional file 1: Appendix 1**. Survey of a nationwide cross-sectional survey of student experiential practice at

community pharmacies in South Korea

1. Which area did you spend most of your time during community pharmacy experiential practice?
   1. Dispensing
   2. Prescription review and computer input
   3. Patient counsel
   4. Nonprescription drugs selection and counsel
   5. Drug Information
   6. Others
2. What was the most preferred task as a pharmacy student trainee?
3. Dispensing
4. Prescription review and computer input
5. Patient counsel
6. Nonprescription drugs selection and counsel
7. Drug Information
8. Others
9. What was the most positive aspect of community pharmacy experiential practice?
10. Help in navigating future career
11. Help in improving professionalism as an entry-level pharmacist
12. Opportunities to learn various areas not taught in school
13. Knowledge gained in school can be linked to real life
14. Was the practice site well-organized and systematically performed?
15. Strongly agree
16. Agree
17. Disagree
18. Strongly disagree
19. Was the experiential practice helpful in future career decision-making ?
20. Strongly agree
21. Agree
22. Disagree
23. Strongly disagree
24. Please respond to the following based on your experience about preceptors’ evaluation while performing community pharmacy experiential practice(CPEP)?

|  | Strongly agree | Agree | Disagree | Strongly disagree |
| --- | --- | --- | --- | --- |
| Criteria for evaluation of the practical training clearly presented |  |  |  |  |
| Your preceptor clearly understand the assessment methods and standards? |  |  |  |  |
| Your preceptor evaluated your practice outcomes fairy |  |  |  |  |

1. Are you planning to undergo elective Advanced Pharmacy Practice Experience (APPE) in a community pharmacy?
2. Yes
3. No
4. Please respond to the following based on your satisfaction of community pharmacy experiential practice (CPEP)?.

|  | Strongly agree | Agree | Disagree | Strongly disagree |
| --- | --- | --- | --- | --- |
| You were satisfied with the CPEP |  |  |  |  |
| The CPEP was rewarding and beneficial to the patients |  |  |  |  |
| The CPEP was a good opportunity to know various fields, such as non-prescription drugs, oriental medicine, medical devices, cosmetics |  |  |  |  |
| The CPEP was an opportunity to link education and practice to improve professionalism |  |  |  |  |
| The CPEP was helpful |  |  |  |  |
| You got a positive impression of community pharmacy after CPEP |  |  |  |  |

1. Please respond to the following based on your experience about stress while performing community pharmacy experiential practice (CPEP)?

|  | Strongly agree | Agree | Disagree | Strongly disagree |
| --- | --- | --- | --- | --- |
| You were stressed out while practicing at the community pharmacy |  |  |  |  |
| You were stressed out by the simple tasks of dispensing |  |  |  |  |
| You were stressed out by the narrow workspace |  |  |  |  |
| You were stressed out by the lack of educational training time due to shortage of staff |  |  |  |  |
| You were stressed out by conflicts between students and the pharmacy staff |  |  |  |  |
| You were stressed out because the entire training was not performed by the preceptor |  |  |  |  |
| You were stressed out by the lack of your capability |  |  |  |  |
| You were stressed out by the lack of preceptors' capability |  |  |  |  |

1. What should be modified to overcome the difficulty in reviewing prescriptions and the need for pharmacist intervention?

|  | Strongly agree | Agree | Disagree | Strongly disagree |
| --- | --- | --- | --- | --- |
| Case-based learning of medication reconciliation |  |  |  |  |
| Accurate knowledge of medications and pharmacotherapeutics |  |  |  |  |
| More training on communication skills |  |  |  |  |
| Motivation and encouragement of students |  |  |  |  |
| Extension of practice period |  |  |  |  |

1. What should be improved in pharmacy practice, especially for preceptors?

|  | Strongly agree | Agree | Disagree | Strongly disagree |
| --- | --- | --- | --- | --- |
| Extension of direct and indirect patient counseling opportunities |  |  |  |  |
| Education on overall pharmacy management |  |  |  |  |
| Reflection of student opinions and feedback |  |  |  |  |
| All practical education to be overseen by preceptors |  |  |  |  |
| Awareness of ethics and the professionalism as a pharmacist |  |  |  |  |

1. What should be modified at the college of pharmacy to improve Experiential Education?
   1. Reflection of student opinions and feedback
   2. Opportunity to select practical sites
   3. Preliminary information about the pharmacy before practice
   4. Sharing and coordination of information between the college of pharmacy & preceptors
   5. Others
2. What should be strengthened in practical training?
   1. Health communication skills with health professionals and patients
   2. Practical education in OTC medication
   3. Education about frequently prescribed medicines
   4. Education about herbal & functional health foods
3. What would you suggest to the college of pharmacy to improve practical training?
4. Sharing and coordinating information on preceptors and schools
5. Reflect feedback from students who completed the practical training
6. Introducing information in advance on the pharmacy for training
7. Granting trainees more options on selecting training site
8. Other
9. What of the following competencies changed through your practical training?

|  | Strongly agree | Agree | Disagree | Strongly disagree |
| --- | --- | --- | --- | --- |
| Identified problems related to the patient’s medications and suggested solutions. |  |  |  |  |
| Monitored the patients’ medication effects, compliance and adverse effects, adjust care plan as needed |  |  |  |  |
| Collected, analyzed, and evaluated subjective and objective patient data, then suggest appropriate pharmacotherapy |  |  |  |  |
| Created, managed, and disposed of pharmacy documents |  |  |  |  |
| Effectively managed the purchase, sale, and inventory of pharmaceuticals and pharmacy items |  |  |  |  |
| Understood the priorities, and carried out tasks systematically |  |  |  |  |
| Counselled and educated patients on pharmacotherapy, non-pharmacotherapy, and preventive therapy |  |  |  |  |
| Provided health education to residents about health promotion, disease prevention, and drug abuse |  |  |  |  |
| Was able to resolve issue by identifying any problems that occurred during the practical training |  |  |  |  |
| Create appropriate presentation materials and was able to deliver the information to the intended audience. |  |  |  |  |
| Empower patients to take responsibility for, and control of, their health |  |  |  |  |
| Advised (motivated) patients to understand and manage their health |  |  |  |  |
| Demonstrate mutual respect (preceptor, colleague) and values of co-operated meet patient care needs |  |  |  |  |
| Effectively communicated with physician and resolve medication-related problems |  |  |  |  |
| Practiced appropriate communication and was able to communicate efficiently |  |  |  |  |
| Experienced and understood the roles and responsibilities of pharmacists |  |  |  |  |
| Realized the importance of improving the competency as a professional through practical training |  |  |  |  |

1. What is your age?

( ) years old

1. What is your gender?
2. Male
3. Female
4. Where was the community pharmacy located that you practiced in?
5. Seoul and Metropolitan
6. Gyeonggi-do
7. Gangwon-do
8. Chungcheong-do
9. Jeolla-do and Jeju-do
10. Gyungsang-do
11. Which was the main factor affecting practice site selection.
12. Transportation time (distance to home)
13. College policy (random assignment)
14. Awareness of pharmacy
15. Others
16. Which of the following items were sold in the practice site?
17. Functional health food
18. Medical devices
19. Animal medicine
20. Cosmetics
21. Oriental medicine
