# Supplementary material for: A nationwide cross-sectional survey of student experiential practice at community pharmacies in South Korea
Source: BMC Med Educ. 2019 Dec 2;19:445. doi: 10.1186/s12909-019-1879-1 (PMC6888916; doi:10.1186/s12909-019-1879-1)
Supplement: Supplementary file 2 — Additional file 2: Appendix 2. Suggestion for improvements in community pharmacy experiential practice [file 12909_2019_1879_MOESM2_ESM.docx]

**Additional file 2: Appendix 2**. Suggestion for Improvements in Community Pharmacy Experiential Practice (n=646)

| **Survey Question**   - Answers | Number (%) |
| --- | --- |
| **What should be modified to overcome student difficulty in reviewing prescriptions and**  **the need for pharmacist intervention?**   - Case-based learning of medication reconciliation - Accurate knowledge of medications and pharmacotherapeutics - More training on communication skills - Motivation and encouragement of students - Extension of practice period | 229 (35.4)  147 (22.8)  142 (22.0)  74 (11.5)  54 (8.4) |
| **What should be improved in pharmacy practice, especially for preceptors?**   - Extension of direct and indirect patient counseling opportunities - Education about overall pharmacy management - Reflection of student opinions and feedback - All practical education to be overseen by preceptors - Awareness of ethics and the sense of being a pharmacist | 311 (48.1)  177 (27.4)  62 (9.6)  49 (7.6)  47 (7.3) |
| **What should be modified at School to improve Experiential Education?**   - Reflection of student opinions and feedback - Opportunities for selection into practical sites - Preliminary information about the pharmacy before practice - Sharing and coordination of information between school & preceptors - Others | 198 (30.7)  184 (28.5)  125 (19.3)  102 (15.8)  37 (5.7) |
| **What do you think is most important to improve practical training?**   - Standardization of experiential education sites - Sharing educational material of students between preceptors - Revision of experiential education manuals to reflect reality - Rotation of practice sites - Extension of practice period | 244 (37.8)  178 (27.6)  149 (23.1)  48 (7.4)  27 (4.2) |
| What should be strengthened in practical training?  Health communication skills, to reflect communication of professionals with patients  Practical education in OTC medication  Education about frequently prescribed medicines  Education about herbal & functional health foods | 260 (40.2)  223 (34.5)  114 (17.6)  49 (7.6) |
